# A retrospective study of the prevalence of isolated prolongation of activated partial thromboplastin time in the preoperative setting

**DOI:** 10.1186/s40981-024-00713-4

**Published:** 2024-05-07

**Authors:** Yasuhiro Watanabe, Yuki Kato, Takayuki Matsuno

**Affiliations:** Department of Anesthesia, Japanese Red Cross Shizuoka Hospital, 8-2 Otemachi Aoi-ku, Shizuoka, 420-0853 Japan

**Keywords:** Activated partial thromboplastin time, Coagulation factor deficiency, Antiphospholipid antibody, Surgery cancellation

## Abstract

**Background:**

Isolated prolongation of activated partial thromboplastin time (APTT) has various causes including inheritable bleeding disorders, and has medical significance as it can lead to the cancelation of surgery. However, even an emergency surgery can be conducted in a patient presenting with severe APTT prolongation, provided careful evaluation and appropriate measures are taken. Hence, the identification of the underlying etiology of the prolonged APTT is crucial. To date, little evidence exists regarding the prevalence of isolated APTT prolongation in Japanese patients undergoing surgery. Herein, we aimed to clarify the prevalence of isolated prolongation of APTT in the preoperative setting and to identify the reasons underlying isolated, severely prolonged APTT.

**Methods:**

Preoperative coagulation data of all elective and emergent patients who presented to the anesthetic department between January 1, 2020, and June 30, 2023, were retrospectively collected. Isolated prolongation of APTT was defined as an APTT ≥ 37 s with an international normalized ratio of prothrombin time < 1.2. The underlying etiology of the patient with isolated, severely prolonged APTT (≥ 46 s) was investigated, and canceled surgical procedures in relation to the isolated APTT prolongation were searched.

**Results:**

Overall, 10,684 measurements from 9413 patients were included, of which 725 (6.8%) were identified as having isolated APTT prolongation. The reasons for the severely prolonged APTT (*n* = 60) were miscellaneous, with the most frequently detected etiology being antiphospholipid antibody positivity. Preoperative isolated APTT prolongation contributed to the cancellation of surgery in elective five cases.

**Conclusions:**

We clarified the prevalence of preoperative isolated prolongation of APTT. The presence of antiphospholipid antibody was the most frequently detected etiology of the patient with isolated, severely prolonged APTT. The present study provides an important dataset regarding the isolated prolongation of APTT in East Asian patients undergoing surgery.

## Background

Routine coagulation tests before surgery are not recommended for unselected patients, since they cannot predict the occurrence of postoperative hemorrhagic complications [1, 2]. However, preoperative coagulation screening still plays a vital role in the detection of coagulopathy, and it can only detect rare bleeding disorders [3]. Activated partial thromboplastin time (APTT), which is clinically utilized to monitor unfractionated heparin and argatroban treatment, detects coagulation factor deficiencies in the intrinsic and common pathway of the coagulation cascade. Moreover, incidental prolongation of APTT can lead to the detection of antiphospholipid antibodies after close examination. Isolated prolongation of APTT has various causes, including pre-analytical artifacts, interference of C-reactive protein, and inheritable bleeding disorders [4–6], and has medical significance as it can lead to the cancellation of surgery. On the other hand, even an emergency surgery utilizing epidural analgesia can be conducted in a patient presenting with severe APTT prolongation, provided careful evaluation and appropriate measures are taken [7]. Thus, it is not uncommon that anesthesiologists encounter preoperative isolated prolongation of APTT and the identification of the underlying etiology of the prolonged APTT is crucial. Although the prevalence of isolated APTT prolongation has been reported [8, 9], limited evidence exists regarding patients undergoing surgery in Japan. The primary aim of this study was to clarify the prevalence of isolated prolongation of APTT in the preoperative setting and to identify the causative factors in patients with isolated, severely prolonged APTT.

## Methods

This single-center retrospective study was approved by the Ethics Committee of the Japanese Red Cross Shizuoka Hospital. In addition to the baseline characteristics, preoperative coagulation data of all elective and emergent patients who presented to the anesthetic department between January 1, 2020, and June 30, 2023, were collected. In principle, we employed the most recent data preceding surgery, in which APTT and the international normalized ratio of prothrombin time (PT-INR) were measured in the same sample. APTT testing was conducted using colloidal silica and synthetic phospholipid as measurement reagents (Coag Genesis APTT, LSI Medience Corporation, Tokyo, Japan) on a fully automated analyzer (STACIA^®^, PHC Corporation, Tokyo, Japan), providing a reference interval of 24–36 s in our institution. Only the measurements during heparin replacement therapy were excluded. Instead, irrespective of the intake of oral anticoagulants and/or antiplatelet drugs, data before initiation and, if available, after the termination of heparin administration were utilized. Isolated prolongation of APTT was defined as an APTT ≥ 37 s accompanied by a PT-INR < 1.2 according to the recent review [9]. The underlying cause of the patient with isolated, severely prolonged APTT (≥ 46 s) was investigated. Finally, canceled surgical procedures in relation to the isolated APTT prolongation were searched.

Continuous variables were expressed as the mean ± standard deviation (SD) unless otherwise mentioned. To compare categorical variables between the groups, the Fisher exact test was performed with the Prism software version 7 (GraphPad Software, La Jolla, CA, US), and a *p* value < 0.05 was considered statistically significant.

## Results

In total, 10,684 measurements from 9413 patients (1–8 samples/patient) were included in this study. Of all 10,684 measurements, 4467 (41.8%) were from male and 6217 (58.2%) were from female patients aged 60.3 ± 20.3 years old. Only 262 (2.5%) measurements were obtained from children under 15 years of age. In addition, 9349 (87.5%) measurements were obtained from patients undergoing elective surgery with the American Society of Anesthesiologists Physical Status (ASA-PS) of 1–4, and the residual 1335 (12.5%) measurements were obtained from patients undergoing emergency surgery with the ASA-PS of 1E–5E.

**Fig. 1:**
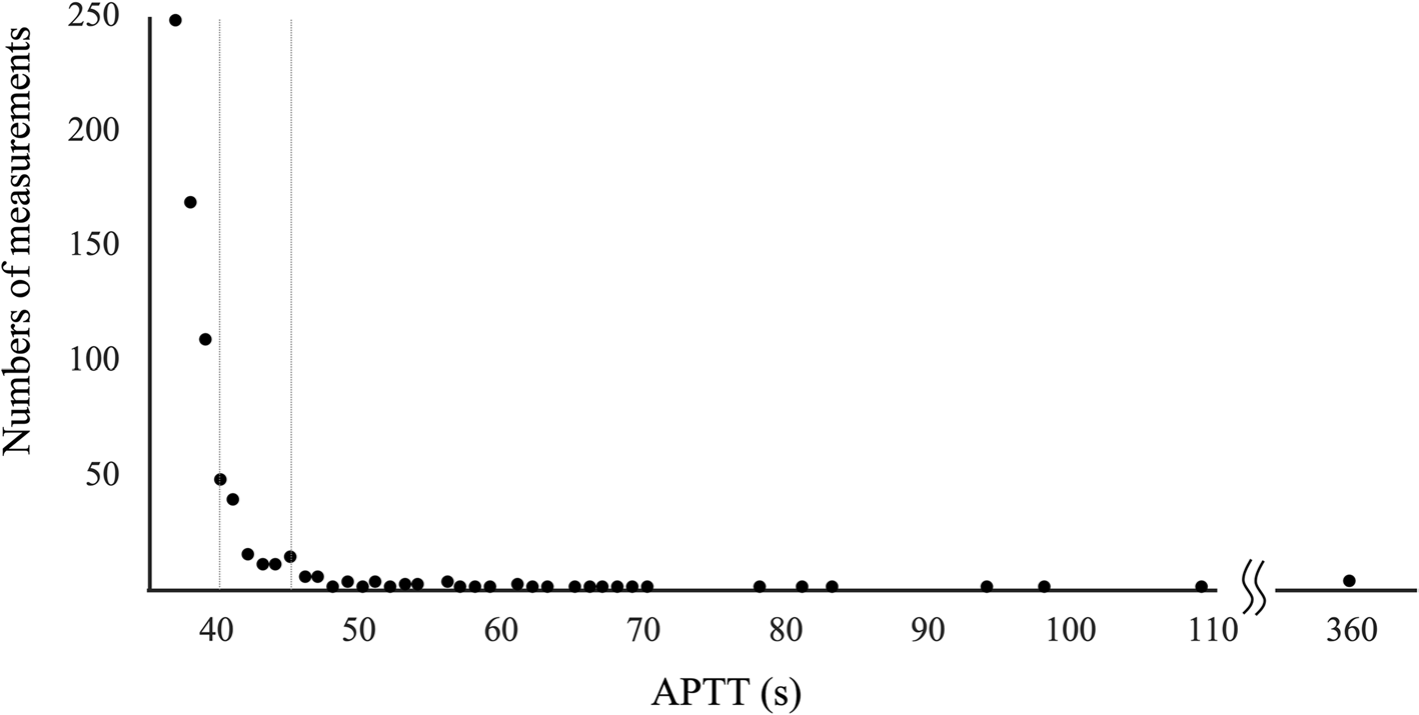
Distribution of values for isolated prolonged APTT. Of the 725 measurements identified as isolated prolongation of APTT, 526 (72.6%) lasted from 37 to 39 s, 139 (19.2%) lasted from 40 to 45 s, and 60 (8.28%) lasted ≥ 46 s. APTT, activated partial thromboplastin time

We identified 725 measurements from 697 patients (1–3 samples/patient) as having isolated prolongation of APTT, corresponding to 6.8% of all 10,684 APTT measurements and 7.2% of the 10,075 measurements with a PT-INR < 1.2 (Table 1). The distribution of values for isolated APTT prolongation is illustrated in Fig. 1. Of the 725 prolonged data, 526 (72.6%) were minimally prolonged (37–39 s), 139 (19.2%) were moderately prolonged (40–45 s), and 60 (8.28%) were severely prolonged (≥ 46 s).

**Table 1:** Numbers of each measurement classified by APTT and PT-INR values

	PT-INR < 1.2	PT-INR ≥ 1.2	Total
**APTT ≤ 36 s**	9350	393	9743
**APTT ≥ 37 s**	725	216	941
**Total**	10,075	609	10,684

A total of 10,684 measurements from 9413 patients were included in our study. Of these, 725 were identified as having isolated prolonged APTT, yielding frequencies of 6.8% of all 10,684 APTT measurements and 7.2% of the 10,075 measurements with a PT-INR < 1.2

*APTT* Activated partial thromboplastin time, *PT-INR* International normalized ratio of prothrombin time

Along with three patients with previously diagnosed antiphospholipid antibody syndrome, thorough hematological investigation, including a cross-mixing study [10], detected positivity for antiphospholipid antibodies as the most frequent etiology of isolated and severely prolonged APTT (*n* = 18). Other determined causes included coagulation factor XII deficiency, coagulation factor VIII deficiency, multiple coagulation factor deficiencies, and hypofibrinogenemia. Several cases exist wherein the etiology has not been examined (*n* = 28). Among these, sepsis or high C-reactive protein level, oral anticoagulant medication, and pre-analytical artifacts were likely responsible in most patients (Table 2). Among these cases presenting with isolated and severely prolonged APTT, surgical patients experienced neither intraoperative massive bleeding nor reexploration for postoperative hemorrhage.

**Table 2:** Etiology and numbers of measurements indicating isolated, severely prolonged APTT

**Etiology**	**Number**
Previously diagnosed or fully investigated	
Antiphospholipid antibody positivity	18
Antiphospholipid antibody syndrome	3
Coagulation factor XII deficiency	3
Coagulation factor VIII deficiency	2
Multiple coagulation factor deficiencies	3
Hypofibrinogenemia	1
Unknown	2
Not investigated	
Sepsis, high C-reactive protein level	11
Oral anticoagulant medication	8
Artifact (very likely)	1
Artifact (possible)	2
Unknown	6
Total	60

Along with three patients with previously diagnosed antiphospholipid antibody syndrome, the thorough hematological investigation revealed the positivity for antiphospholipid antibodies as the most frequent etiology of isolated, severely prolonged APTT (≥ 46 s). Other determined causes included coagulation factor XII deficiency, coagulation factor VIII deficiency, multiple coagulation factor deficiencies, and hypofibrinogenemia. Several cases exist wherein the etiology has not been examined. Among these, sepsis or high C-reactive protein level, oral anticoagulant medication, and pre-analytical artifacts were considered to be responsible for most patients

*APTT* Activated partial thromboplastin time

Surgery was canceled in elective five cases after a close examination of the isolated prolonged APTT. The causative factors of APTT prolongation and the APTT values in each of the five cases were antiphospholipid antibody positivity (50 s), coagulation factor XII deficiency (63 s), coagulation factor VIII deficiency (44 s), hypofibrinogenemia (61 s), and unknown etiology despite thorough investigation (66 s).

## Discussion

To the best of our knowledge, this is the largest retrospective study addressing the prevalence of isolated APTT prolongation in Japanese surgical patients. In our study, 725 (6.8%) of 10,684 measurements indicated isolated APTT prolongation.

Although routine coagulation screening tests prior to surgery are not recommended, it has been suggested that preoperative history taking without hematologic evaluation may underestimate the prevalence of bleeding disorders, leading to perioperative morbidity and mortality [11]. In our practice, preoperative coagulation tests were conducted for all patients who presented to the anesthetic department, contributing to the elimination of selection bias.

The frequency of isolated prolongation of APTT in our study was considerably lower than that recently reported (i.e., isolated prolongation of APTT ≥ 38 s corresponded to 12.1% of the APTT measurements accompanied by a normal prothrombin time and/or PT-INR < 1.2) [9]. The Danish study investigated 18,642 samples from 10,697 patients (1–56 samples/patient), suggesting that multiple samples were collected from a significant number of patients. In our cohort, multiple measurements were confirmed in 2327 samples from 1056 patients (i.e., 2–8 samples from one patient), and the residual 8357 measurements were sampled from the same number of 8357 patients. It can be postulated that data collection differences may have resulted in a discrepancy with our results. In contrast, another Italian retrospective study demonstrated that 463 (5.74%) of 8069 adults undergoing elective surgery had prolonged APTT [8], which is comparable to our data. In our present study, isolated prolongation of APTT was detected more frequently in the measurements of patients undergoing elective surgery compared with those undergoing emergency surgery (657 [7.03%] of 9349 measurements vs 68 [5.09%] of 1335 measurements; *p* < 0.05). The exact reason is unclear; however, it is possible that emergency surgeries had been deferred or even canceled because of severe coagulation abnormalities before they were presented to the anesthetic department.

Special attention should be paid to the five cases wherein surgery was canceled due to isolated APTT prolongation. All but one patient demonstrated severely prolonged APTT. The patients with antiphospholipid antibody positivity and coagulation factor XII deficiency did not demonstrate bleeding tendencies [12]; nevertheless, surgeries were canceled entirely at the surgeon’s discretion. The others with coagulation factor VIII deficiency, hypofibrinogenemia [13], and unknown etiology exhibited a prolonged bleeding time and clinical signs of bleeding tendency. Particularly, in the case with hypofibrinogenemia, the transfusion of fresh frozen plasma minimally contributed to the increase in plasma fibrinogen levels. Thus, the decision to cancel surgery was based on a multidisciplinary evaluation of the patient.

The present study had several limitations. First, this study was a single-center study, and the measurements were mostly obtained from adult patients. Our findings should be interpreted within the context; however, we believe that our large-scale investigation provided a significant dataset regarding the isolated APTT prolongation in Japanese patients undergoing surgery. Second, due to the retrospective nature of our study, the etiology was not fully elucidated in patients with severely prolonged APTT. Practically, investigating every patient presenting with an isolated, severely prolonged APTT was challenging, as not all such patients had the time to afford scrutiny. However, our finding that the presence of antiphospholipid antibodies was the most frequently detected etiology was partly consistent with previously reported results [14–16], supporting the validity of our data.

## Conclusions

We clarified the prevalence of preoperative isolated prolongation of APTT. Our findings suggest that isolated APTT prolongation requires anesthesiologists to determine the feasibility of the surgical procedure based on the evaluation of bleeding tendency along with other hemostatic values. This study provides an important dataset regarding the isolated prolongation of APTT in East Asian patients undergoing surgery.

## Data Availability

All relevant data used to support the findings of this study are included in this article. Other datasets are available from the corresponding author upon request.

## Abbreviations

APTT: Activated partial thromboplastin time
ASA-PS: American Society of Anesthesiologists Physical Status
PT-INR: International normalized ratio of prothrombin time

## References

[1] van Veen JJ, Spahn DR, Makris M (2011). Routine preoperative coagulation tests: an outdated practice?. Br J Anaesth.

[2] Levy JH, Szlam F, Wolberg AS, Winkler A (2014). Clinical use of the activated partial thromboplastin time and prothrombin time for screening. Clin Lab Med.

[3] Jonnavithula N, Durga P, Pochiraju R, Anne KK, Ramachandran G (2009). Routine preoperative coagulation screening detects a rare bleeding disorder. Anesth Analg.

[4] Kitchens CS (1988). Prolonged activated partial thromboplastin time of unknown etiology: a prospective study of 100 consecutive cases referred for consultation. Am J Hematol.

[5] Santoro RC, Molinari AC, Leotta M, Martini T (2023). Isolated prolongation of activated partial thromboplastin time: not just bleeding risk!. Medicina (Kaunas).

[6] Liu J, Li F, Shu K, Chen T, Wang X, Xie Y (2018). The analysis of false prolongation of the activated partial thromboplastin time (activator: silica): interference of C-reactive protein. J Clin Lab Anal.

[7] Watanabe Y, Kaneda T (2022). Anesthetic management using epidural analgesia for emergency laparoscopic cholecystectomy in a patient with lupus anticoagulant positivity and prolonged activated partial thromboplastin time. Case Rep Anesthesiol.

[8] Tagariello G, Radossi P, Salviato R, Zardo M, De Valentin L, Basso M (2017). Clinical relevance of isolated prolongation of the activated partial thromboplastin time in a cohort of adults undergoing surgical procedures. Blood Transfus.

[9] Rasmussen KL, Philips M, Tripodi A, Goetze JP (2020). Unexpected, isolated activated partial thromboplastin time prolongation: a practical mini-review. Eur J Haematol.

[10] Kamal AH, Tefferi A, Pruthi RK (2007). How to interpret and pursue an abnormal prothrombin time, activated partial thromboplastin time, and bleeding time in adults. Mayo Clin Proc.

[11] Bitar M, Dunya G, Khalifee E, Muwakkit S, Barazi R (2019). Risk of post-operative hemorrhage after adenoidectomy and tonsillectomy: value of the preoperative determination of partial thromboplastin time and prothrombin time. Int J Pediatr Otorhinolaryngol.

[12] Mishra L, Lee D, Ho KM (2023). Incidence of factor XII deficiency in critically ill patients with a prolonged activated partial thromboplastin time: a prospective observational study. Blood Coag Fibrinol.

[13] Richard M, Celeny D, Neerman-Arbez M (2022). Mutations accounting for congenital fibrinogen disorders: an update. Semin Thromb Hemost.

[14] Chng WJ, Sum C, Kuperan P (2005). Causes of isolated prolonged activated partial thromboplastin time in an acute care general hospital. Singapore Med J.

[15] Hazim AZ, Ruan GJ, Khodadadi RB, Slusser JP, Marshall AL, Pruthi RK (2022). A single-institution retrospective study of causes of prolonged prothrombin time and activated partial thromboplastin time in the outpatient setting. Int J Lab Hematol.

[16] Barbosa ACN, Montalvão SAL, Barbosa KGN, Colella MP, Annichino-Bizzacchi JM, Ozelo MC (2019). Prolonged APTT of unknown etiology: a systematic evaluation of causes and laboratory resource use in an outpatient hemostasis academic unit. Res Pract Thromb Haemost.

